# Functional outcome, in-hospital healthcare consumption and in-hospital costs for hospitalised traumatic brain injury patients: a Dutch prospective multicentre study

**DOI:** 10.1007/s00701-020-04384-9

**Published:** 2020-05-14

**Authors:** Jeroen T. J. M. van Dijck, Cassidy Q. B. Mostert, Alexander P. A. Greeven, Erwin J. O. Kompanje, Wilco C. Peul, Godard C. W. de Ruiter, Suzanne Polinder

**Affiliations:** 1 Department of Neurosurgery, University Neurosurgical Center Holland, LUMC, HMC & Haga Teaching Hospital, Leiden, The Hague The Netherlands; 2 LUMC, Albinusdreef 2, J-11-R-83, 2333 ZA Leiden, The Netherlands; 3 Department of Surgery, Haga Teaching Hospital, The Hague, The Netherlands; 4 Department of Intensive Care, Erasmus MC—University Medical Centre Rotterdam, Rotterdam, The Netherlands; 5 Department of Medical Ethics and Philosophy of Medicine, Erasmus MC—University Medical Centre Rotterdam, Rotterdam, The Netherlands; 6 Department of Public Health, Erasmus MC—University Medical Centre Rotterdam, Rotterdam, The Netherlands

**Keywords:** Traumatic brain injury, In-hospital costs, Mortality, Functional outcome

## Abstract

**Background:**

The high occurrence and acute and chronic sequelae of traumatic brain injury (TBI) cause major healthcare and socioeconomic challenges. This study aimed to describe outcome, in-hospital healthcare consumption and in-hospital costs of patients with TBI.

**Methods:**

We used data from hospitalised TBI patients that were included in the prospective observational CENTER-TBI study in three Dutch Level I Trauma Centres from 2015 to 2017. Clinical data was completed with data on in-hospital healthcare consumption and costs. TBI severity was classified using the Glasgow Coma Score (GCS). Patient outcome was measured by in-hospital mortality and Glasgow Outcome Score–Extended (GOSE) at 6 months. In-hospital costs were calculated following the Dutch guidelines for cost calculation.

**Results:**

A total of 486 TBI patients were included. Mean age was 56.1 ± 22.4 years and mean GCS was 12.7 ± 3.8. Six-month mortality (4.2%–66.7%), unfavourable outcome (GOSE ≤ 4) (14.6%–80.4%) and full recovery (GOSE = 8) (32.5%–5.9%) rates varied from patients with mild TBI (GCS13–15) to very severe TBI (GCS3–5). Length of stay (8 ± 13 days) and in-hospital costs (€11,920) were substantial and increased with higher TBI severity, presence of intracranial abnormalities, extracranial injury and surgical intervention. Costs were primarily driven by admission (66%) and surgery (13%).

**Conclusion:**

In-hospital mortality and unfavourable outcome rates were rather high, but many patients also achieved full recovery. Hospitalised TBI patients show substantial in-hospital healthcare consumption and costs, even in patients with mild TBI. Because these costs are likely to be an underestimation of the actual total costs, more research is required to investigate the actual costs-effectiveness of TBI care.

**Electronic supplementary material:**

The online version of this article (10.1007/s00701-020-04384-9) contains supplementary material, which is available to authorized users.

## Introduction

Recent estimates indicate that worldwide up to 69 million people a year sustain a traumatic brain injury (TBI). [15] The high incidence of TBI and the associated acute and chronic sequelae cause substantial healthcare and socio-economic challenges. [32] Available treatments are unfortunately still largely unproven or unsatisfactory. [9, 15, 32, 75] Patients suffer from the medical consequences of TBI, which range from headache and fatigue to severe disabilities and even death [4, 14, 18, 59, 68]. The total global accompanying costs of around US$ 400 billion a year are a major challenge from a socioeconomic perspective [32], especially considering the fact that TBI-related healthcare costs are rising, while healthcare budgets remain limited [19]. The in-hospital costs related to TBI represent a substantial part of the total utilised resources [49]. Unfortunately, understanding and generalising the in-hospital costs of individual TBI patients from available literature remains difficult because methodological heterogeneity of TBI cost studies is high and study quality often inadequate [1, 30, 69].

Accurate insight in TBI-related costs is essential to substantiate research initiatives that aim to improve treatment efficiency. It also guides policymakers on the rational allocation of resources without compromise of patient outcome. To allow healthcare professionals to continue to provide optimal care for their patients, high-quality cost-analysis studies are urgently needed [1, 30].

Therefore, the aim of this study is to describe outcome, in-hospital healthcare consumption and in-hospital costs of hospitalised TBI patients.

## Materials and methods

This study followed the recommendations from the ‘Strengthening the Reporting of Observational Studies in Epidemiology’ STROBE statement [76].

### Study design and patients

Patients were included in three level 1 trauma hospitals from January 2015 to September 2017. All hospitals are located in an urban area in the mid-Western part of the Netherlands and participated in the Collaborative European NeuroTrauma Effectiveness Research in Traumatic Brain Injury (CENTER-TBI) project. The CENTER-TBI Core study (clinicaltrials.gov NCT02210221; RRID: SCR_015582) is a prospective multicentre longitudinal observational study conducted in 65 centres across Europe and Israel [31]. The project aimed to improve TBI characterisation and classification and to identify best clinical care. The responsible institutional review board (METC Leiden) approved this study (P14.222).

Patients were included in the CENTER-TBI Core study using the following criteria: (1) clinical diagnosis of TBI, (2) clinical indication for head CT scan, (3) presentation to study centre within 24 h after injury and (4) informed consent following Dutch requirements, including patient, proxy and deferred consent. Patients were excluded when they had a severe pre-existing neurological disorder that would confound outcome assessments or in case of insufficient understanding of the Dutch or English language.

### Clinical data

Clinical data were prospectively collected by using a web-based electronic case report form (CRF) (QuesGen System Incorporated, Burlingame, CA, USA). Data were obtained from electronic patient files and patient interviews and when necessary initially recorded on a hardcopy CRF. Data collection was completed by a local research staff that was specifically trained for this project. The site’s principal investigator supervised the project. Data were de-identified by using a randomly generated GUPI (Global Unique Patient Identifier) and was stored on a secure database, hosted by the International Neuroinformatics Coordinating Facility (INCF; www.incf.org) in Stockholm, Sweden.

Data was extracted in December 2019 (version 2.1) using a custom-made data access tool Neurobot (http://neurobot.incf.org), developed by INCF (RRID: SCR_01700). Extracted data included baseline demographic, trauma and injury information, results of neurological assessments, imaging (first head CT scan) and patient outcome. This database was merged with separately collected data on in-hospital healthcare consumption and in-hospital costs, which is explained later. Discrepancies were resolved by source data verification.

Baseline Glasgow Coma Scale (GCS) Total Score, GCS Motor Score and pupillary reactivity variables were collected. TBI severity was then classified by using the GCS (GCS13–15; mild TBI, GCS9–12; moderate TBI, GCS3–8; severe TBI, GCS3–5; very severe TBI) [64]. These values were derived variables that were centrally calculated using the IMPACT methodology, taking a post stabilisation value and if absent work back in time towards prehospital values. Out of 19 missing GCS values, 8 were completed by using emergency department arrival GCS score. Intubation was calculated as a GCS verbal score of 1. Major extracranial injury was defined by AIS body region ≥ 3. Characteristics from the first head CT scan were assessed by a central review panel [73]. Six out of seven missing central assessments were completed by using the assessments of local radiologists. Outcome data included in-hospital mortality and 6-month Glasgow Outcome Score–Extended (GOSE). GOSE outcome was dichotomised in favourable (GOSE ≥ 5) and unfavourable (GOSE ≤ 4) [78].

### In-hospital healthcare consumption

We collected in-hospital healthcare consumption data from electronic patient records by using a predefined cost assessment database. The Dutch National Health Care Institute Guidelines for healthcare cost calculation were followed [23]. Units (e.g. number of admission days, number of diagnostics) were collected independently by two researchers from the electronic patient files. There were five main categories: (1) admission; including length of stay (LOS) in (non-)ICU with consultations, (2) surgical interventions, (3) imaging, (4) laboratory; including blood products and (5) other; including ambulance transportation and outpatient visits [70]. Non-ICU admission was defined as admission to a ward or medium care. In-hospital healthcare consumption and costs were calculated for all included patients (Supplement 1).

### In-hospital costs

We focused on the in-hospital costs from a healthcare perspective. Costs of re-admissions and costs of visits to the Outpatient Clinic related to the trauma were also included. The methods and reference prices as described in the Dutch Guidelines for economic healthcare evaluations were used to calculate in-hospital costs [23]. Costs were calculated by multiplying the number of consumed units with the corresponding guideline reference price. Guideline reference prices are based on non-site specific large patient cohorts which improves their generalisability and interpretation [23]. When reference prices were not mentioned, the remaining units were valued by using amounts per unit as reported by The Netherlands Healthcare Authority (NZa) (i.e. diagnostics) [83] or by using their average national price, based on declared fees (i.e. surgical interventions, consultations) [82]. All costs were converted to the last year of patient inclusion (2017) using the national general consumer price index (CBS) and rounded to the nearest ten euros. One EURO equalled $1.05 dollar on the 1st of January 2017 (Supplement 1).

### Statistical methods

Data were analysed using descriptive statistics. Baseline data were presented as absolute numbers and percentages. Continuous variables, like LOS and costs, were presented as mean ± standard deviation or median (interquartile range 25–75). Subgroups were made using age, TBI severity, pupillary abnormalities, intracranial abnormalities, surgical intervention and outcome. ANOVA and χ^2^ were used for comparison of continuous and categorical variables across different subgroups. A *p* value of < 0.05 was considered statistically significant. All analyses were performed using IBM’s statistical package for social sciences version 25.0 (SPSS). Figures were designed using GraphPad Prism 8.

## Results

A total of 486 patients with TBI were included in this study. Patients had a mean age of 56.1 ± 22.4 years and were predominantly male (60.5%) (Table 1). Nearly all patients sustained a closed head injury (98.4%). TBI was mainly caused by incidental falls (54.3%) or road traffic accidents (36.2%) and occurred on streets (56.2%) or at home (31.5%). The mean baseline GCS was 12.7 ± 3.8 and mean injury severity score (ISS) was 20 ± 16. Patients sustained mild TBI (*N* = 354, 72.8%), moderate TBI (*N* = 43, 8.8%) and severe TBI (*N* = 78, 16.1%), of which 51 were very severe (10.5%). Loss to follow-up was 14.2% and not significantly different between severity groups.

**Table 1:** Patient characteristics and outcome

	All (*N* = 486)	Mild TBI (*N* = 354)	Moderate TBI (*N* = 43)	Severe TBI (*N* = 78)	Very severe TBI (*N* = 51)	*p* value*
Male	294 (60.5)	211 (59.6)	25 (58.1)	54 (69.2)	36 (70.6)	0.265
Age (years)	56.1 ± 22.4	56.6 ± 22.2	58.5 ± 22.4	52.2 ± 22.6	50.9 ± 23.3	0.222
≤ 18	25 (5.1)	21 (5.9)	1 (2.3)	2 (2.6)	2 (3.9)	0.467
19–64	255 (52.5)	184 (52.0)	21 (48.8)	46 (59.0)	30 (58.8)	
≥ 65	206 (42.4)	149 (42.1)	21 (48.8)	30 (38.5)	19 (37.3)	
Stratum						< 0.001
Admission	319 (65.6)	288 (81.4)	16 (37.2)	9 (11.5)	5 (9.8)	
ICU	167 (34.4)	66 (18.6)	27 (62.8)	69 (88.5)	46 (90.2)	
Location of injury						0.137
Street/highway	273 (56.2)	201 (56.8)	22 (51.2)	45 (57.7)	31 (60.8)	
Home/domestic	153 (31.5)	113 (31.9)	11 (25.6)	25 (32.1)	15 (29.4)	
Work/school	14 (2.9)	8 (2.3)	5 (11.6)	1 (1.3)	1 (2.0)	
Sport/recreational	18 (3.7)	14 (4.0)	2 (4.7)	1 (1.3)	0 (0.0)	
Public location	25 (5.1)	15 (4.2)	3 (7.0)	6 (7.7)	4 (7.8)	
Other/unknown	2 (0.6)	3 (0.9)	0 (0.0)	0 (0)	0 (0.0)	
Cause of injury						0.136
Road traffic accident	176 (36.2)	125 (35.3)	14 (32.6)	35 (44.9)	25 (49.0)	
Incidental fall	264 (54.3)	200 (56.5)	21 (48.8)	35 (44.9)	20 (39.2)	
Non-intentional injury	12 (2.5)	8 (2.3)	2 (4.7)	1 (1.3)	1 (2.0)	
Violence/assault	10 (2.1)	8 (2.3)	2 (4.7)	0 (0.0)	0 (0.0)	
Suicide attempt	3 (0.6)	0 (0.0)	1 (2.3)	2 (2.6%)	2 (3.9)	
Other/unknown	21 (4.3)	13 (3.6)	3 (7.0)	5 (6.4)	3 (5.9)	
Glasgow Coma Score	12.7 ± 3.8	14.7 ± 0.6	10.6 ± 0.9	4.7 ± 1.9	3.5 ± 0.7	N/A
GCS Motor score	5.3 ± 1.6	6.0 ± 0.4	5.0 ± 1.3	2.3 ± 1.7	1.4 ± 0.8	
GCS 13–15	354 (72.8)	354 (100)	–	–	–	
GCS 9–12	43 (8.8)	–	43 (100)	–	–	
GCS 3–8	78 (16.1)	–	–	78 (100)	–	
GCS 3–5	51 (10.5)	–	–	51 (65.4)	51 (100)	
Missing	11 (2.3)	–	–	–	–	
Pupillary abnormalities						< 0.001
Both reacting	423 (87.0)	343 (98.0)	39 (90.7)	38 (48.7)	19 (37.3)	
One reacting	14 (2.9)	5 (1.4)	2 (4.7)	7 (9.0)	4 (7.8)	
Both non-reacting	37 (7.6)	2 (0.6)	2 (4.7)	33 (42.3)	28 (54.9)	
Missing	12 (2.5)	4 (1.1)	0 (0.0)	0 (0.0)	0 (0.0)	
Findings first CT scan						
Intracranial abnormalities	263 (54.1)	160 (45.2)	30 (69.8)	68 (87.2)	43 (84.3)	< 0.001
Contusion	130 (26.7)	68 (19.2)	22 (51.2)	38 (48.7)	26 (51.0)	< 0.001
Traumatic SAH	185 (38.1)	101 (28.5)	26 (60.5)	56 (71.8)	37 (72.5)	< 0.001
Epidural hematoma(s)	47 (9.7)	27 (7.6)	7 (16.3)	13 (16.7)	9 (17.6)	< 0.001
Subdural hematoma(s)	136 (28.0)	68 (19.2)	22 (51.2)	43 (55.1)	28 (54.9)	< 0.001
Skull fracture(s)	180 (37.0)	97 (27.4)	25 (58.1)	55 (70.5)	39 (76.5)	< 0.001
Compressed basal cisterna	88 (18.1)	30 (8.5)	9 (20.9)	47 (60.3)	34 (66.7)	< 0.001
Midline shift > 5 mm	65 (13.4)	21 (5.9)	10 (23.3)	31 (39.7)	20 (39.2)	< 0.001
Mass lesion > 25 cc	80 (16.5)	26 (7.3)	14 (32.6)	37 (47.4)	26 (51.0)	< 0.001
Uninterpretable**	10 (2.1)	5 (1.4)	4 (9.3)	0 (0.0)	0 (0.0)	
Injury severity						
Brain Injury AIS	3.1 ± 1.2	2.7 ± 0.9	3.7 ± 1,2	4.6 ± 1.2	4.8 ± 1.2	< 0.001
ISS	20 ± 16	15 ± 9	22 ± 16	39 ± 22	43 ± 21	< 0.001
In-hospital mortality	60 (12.3)	8 (2.3)	8 (18.6)	42 (53.8)	32 (62.7)	< 0.001
GOSE at 6 months	5.72 ± 2.55	6.5 ± 1.8	4.6 ± 2.7	2.9 ± 2.7	2.4 ± 2.5	
Favourable/unfavourable***	72.9%/27.1%	85.4%/14.6%	55.3%/44.7%	29.0%/71.0%	19.6%/80.4%	< 0.001
1	73 (15.0)	15 (4.2)	10 (23.3)	45 (57.7)	34 (66.7)	< 0.001
2/3	17 (3.5)	10 (2.8)	6 (14.0)	1 (1.3)	0 (0.0)	
4	23 (4.7)	19 (5.4)	1 (2.3)	3 (3.8)	3 (5.9)	
5	25 (5.1)	18 (5.1)	5 (11.6)	2 (2.6)	1 (2.0)	
6	38 (7.8)	31 (8.8)	4 (9.3)	3 (3.8)	1 (2.0)	
7	110 (22.6)	93 (26.3)	4 (9.3)	10 (12.8)	4 (7.8)	
8	131 (27.0)	115 (32.5)	8 (18.6)	5 (6.4)	3 (5.9)	
Loss to follow-up	69 (14.2)	53 (15.0)	5 (11.6)	9 (11.5)	5 (9.8)	0.650

Values are reported as: Number (percentage). Mean ± SD. *AIS*, abbreviated injury scale; *CT scan*, computed tomography scan; *GCS*, Glasgow Coma Score; *GOSE*, Glasgow Outcome Score–Extended; *ICU*, intensive care unit; *SAH*, subarachnoid haemorrhage

^*^p values were derived from ANOVA for continuous characteristics and χ^2^ statistics for categorical characteristics, comparing TBI severity categories (severe TBI, moderate TBI, mild TBI). The *p* value assessed compatibility with the null hypothesis of no differences between TBI severity categories

^**^Numbers from TBI severity subgroups do not always match the numbers that are reported for all patients because baseline GCS data was missing for 11 patients. Also, data from 1 CT scan could not be retrieved

^***^Calculated excluding missing. Favourable and unfavourable were defined as GOSE 5–8 and GOSE 1–4 respectively

### Patient outcome

**Fig. 1:**
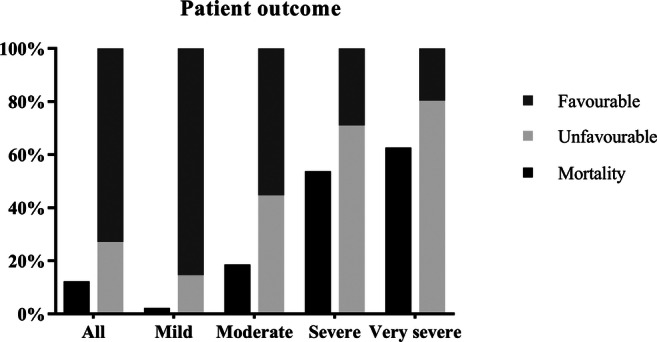
In-hospital mortality and functional outcome (favourable GOSE 5–8, unfavourable GOSE 1–4) at 6 month follow-up for patients with TBI in different severities

Mean in-hospital mortality was 12.3% and ranged from 2.3% for patients with mild TBI to 62.7% for patients with very severe TBI (Table 1). The 6-month GOSE follow-up was available for 417 patients (85.8%). Favourable outcome (GOSE ≥ 5) was achieved by 85.4% of patients with mild, 55.3% with moderate, 29.0% with severe and 19.6% with very severe TBI (Fig. 1). A GOSE of 2–4 was found in 40 survivors (8.2%), of which 17 (3.5%) were in a vegetative state (GOSE = 2) or required full assistance in daily life (GOSE = 3). Nearly a third of patients reported full recovery (GOSE = 8) after mild (32.5%), 18.6% after moderate, 6.4% after severe and 5.9% after very severe TBI.

### Length of stay and surgical interventions

**Fig. 2:**
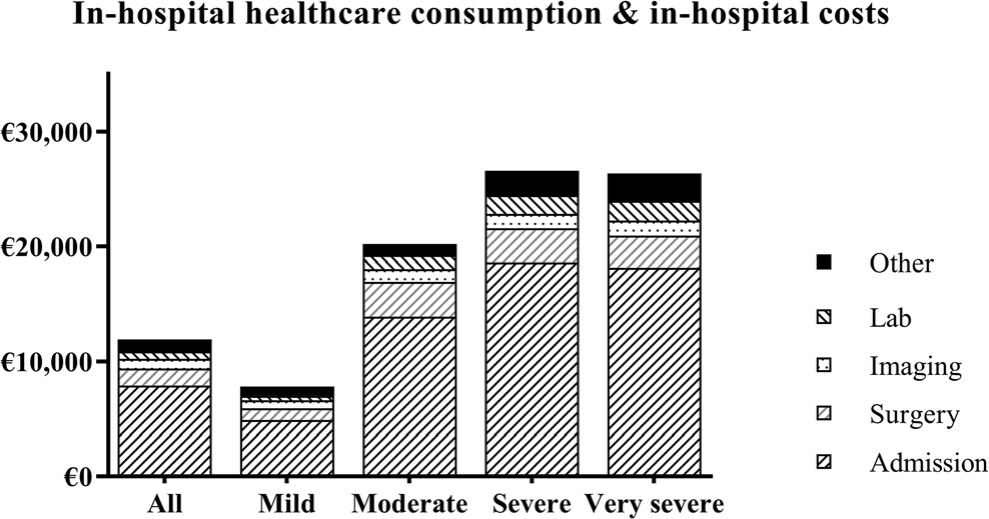
The mean in-hospital costs for patients with TBI, specified per severity category and per cost category to show their contribution to the total in-hospital costs

Mean total LOS was 8 days (2 days on ICU and 6 days non-ICU). LOS significantly increased with TBI severity, presence of major extracranial injury, surgical intervention(s) and presence of all types of intracranial abnormalities except epidural hematoma (Table 2, Fig. 2). Patients that required ICP monitoring and/or a decompressive craniectomy showed longest mean LOS (27 and 28 days respectively). LOS was short in patients without intracranial abnormalities (5 days). Patients with two non-reacting pupils also showed a significantly shorter LOS (5 days) compared with those with either one (17 days) or two reacting pupils (8 days).

**Table 2:** Length of stay and in-hospital costs

Patient category	N	Total LOS	ICU LOS	Non-ICU LOS	Total costs	Admission costs	Surgery costs	Radiology costs	Laboratory costs
All patients	486	8 ± 13	2 ± 5	6 ± 10	11,920; 5200 (2780-12,500)	7900; 2670 (1430-7090)	1490; 0 (0–1820)	840; 670 (350–1080)	650; 130 (59–580)
Age								*	
≤ 18	25	3 ± 4	1 ± 4	2 ± 2	6100; 2550 (1830–6470)	4110; 1840 (1180-2600)	650; 0 (0–0)	460; 300 (130–440)	210; 50 (0–70)
19–64	255	8 ± 15	2 ± 5	6 ± 11	12,640; 4560 (2720-12,630)	8230; 2440 (1370-6810)	1760; 0 (0–3160)	900; 780 (370–1160)	620; 100 (60–470)
≥ 65	206	8 ± 11	2 ± 5	7 ± 8	11,720; 6240 (3070-13,060)	7940; 3800 (1840-7620)	1270; 0 (0–0)	810; 650 (350–980)	740; 200 (70–780)
TBI severity		*	*	*	*	*	*	*	*
GCS 13–15	354	6 ± 8	1 ± 3	5 ± 6	7800; 3880 (2550-8630)	4900; 2050 (1430-5250)	1000; 0 (0–0)	720; 570 (310–930)	330; 80 (60–240)
GCS 9–12	43	14 ± 15	4 ± 6	10 ± 12	20,210; 12,480 (5370-27,220)	13,900; 8680 (2500-18,910)	3010; 0 (0–4520)	1140; 890 (480–1560)	1170; 570 (160–1820)
GCS 3–8	78	15 ± 22	6 ± 9	9 ± 18	26,600; 12,340 (7730-41,260)	18,630; 6570 (2670-26,410)	2950; 0 (0–4520)	1240; 980 (720–1650)	1660; 730 (240–2550)
GCS 3–5	51	14 ± 20	6 ± 8	7 ± 17	26,350; 12,500 (7730-42,430)	18,140; 6230 (2670-30,600)	2790; 0 (0–4530)	1310; 1010 (760–1940)	1730; 790 (240–2980)
Pupil reactivity		*	*	*	*	*	*		*
Both reacting	423	8 ± 13	2 ± 5	6 ± 10	11,270; 4650 (2700-12,290)	7540; 2600 (1430-7070)	1400; 0 (0–0)	830; 650 (340–1070)	560; 110 (60–480)
One reacting	14	17 ± 16	8 ± 11	9 ± 7	31,940; 13,600 (5070-51,490)	22,330; 6420 (2890-33,050)	4210; 3840 (0–7440)	1250; 1290 (290–2260)	2330; 1120 (370–4480)
None reacting	37	5 ± 6	3 ± 5	2 ± 5	13,210; 8210 (6220-14,060)	7570; 2670 (2340-7210)	1800; 0 (0–4520)	880; 840 (660–1010)	1160; 570 (210–1230)
Early CT scan									
Yes abnormalities	263	10 ± 15*	3 ± 6*	7 ± 11*	15,780; 8240 (3690-15,750)*	10,830; 4340 (1880-10,290)*	1860; 0 (0–3720)*	930; 760 (400–1190)*	940; 240 (70–1080)*
No abnormalities	212	5 ± 8	0 ± 2	4 ± 7	6490; 3180 (2350-6670)	3860; 1840 (1180-3950)	870; 0 (0–0)	700; 500 (290–920)	260; 70 (60–190)
Contusion	139	12 ± 16*	3 ± 6*	8 ± 13*	18,060; 9810 (4100-21,560)*	12,740; 5580 (2340-15,670)*	2190; 0 (0–3720)*	970; 800 (500–1210)*	1010; 370 (70–1230)*
Traumatic SAH	185	11 ± 17*	3 ± 7*	8 ± 13*	17,730; 9090 (4130-20,640)*	12,250; 4930 (2340-13,520)*	2120; 0 (0–4520)*	990; 840 (450–1280)*	1080; 400 (80–1280)*
Epidural hematoma(s)	47	10 ± 15	3 ± 6	8 ± 11	16,320; 8240 (3170-14,060)	11,390; 4670 (1840-11,520)	1980; 0 (0–1820)	910; 790 (400–1140)	720; 220 (60–710)
Subdural hematoma(s)	136	11 ± 16*	3 ± 6*	8 ± 12*	16,670; 8800 (4210-20,290)*	11,180; 4680 (1880-13,170)*	2290; 0 (0–4520)	950; 790 (460–1200)*	1100; 410 (100–1350)*
Skull fracture(s)	180	9 ± 15*	3 ± 6*	7 ± 11	15,450; 8190 (3350-16,560)*	10,620; 4140 (1970-12,300)*	1730; 0 (0–3160)	900; 770 (400–1190)	900; 240 (60–1070)*
Compressed basal cisterna	88	12 ± 18*	4 ± 7*	8 ± 13	21,000; 10,520 (6500-26,030)*	13,890; 5710 (2670-17,210)*	3190; 1580 (0–4520)*	1080; 860 (590–1520)*	1460; 570 (200–1930)*
Midline shift > 5 mm	65	12 ± 15*	4 ± 7*	8 ± 12	21,290; 12,410 (6810-26,440)*	13,950; 6530 (2670-16,940)*	3630; 4520 (0–4530)*	1050; 820 (570–1480)*	1420; 770 (240–1910)*
Mass lesion > 25 cc	80	12 ± 18*	5 ± 8*	8 ± 13	21,590; 11,840 (6960-25,230)*	14,620; 6630 (2670-15,060)*	3230; 3530 (0–4520)*	1120; 840 (590–1540)*	1420; 560 (220–1520)*
Surgical intervention									
Intracranial surgery	67	21 ± 23*	8 ± 9*	13 ± 18*	36.870; 26,440 (13,210-48,500)*	24,970; 15,560 (6740-33,050)*	6670; 4530 (4520-8250)*	1510; 1230 (840–2100)*	2300; 1480 (570–4280)*
No intracranial surgery	419	6 ± 8	1 ± 4	5 ± 7	7930; 4110 (2600-8960)	5170; 2400 (1430-5300)	670; 0 (0–0)	730; 600 (310–960)	390; 90 (60–300)
ICP monitoring	40	27 ± 28*	12 ± 9*	16 ± 22*	47,260; 41,850 (21,480-63,500)*	33,670; 26,530 (13,100-50,180)*	7220; 5430 (4520-8250)*	1690; 1710 (870–2310)*	2880; 1960 (1040-4780)*
No ICP monitoring	446	6 ± 9	1 ± 4	5 ± 7	8750; 4510 (2640-10,900)	5590; 2500 (1430-5840)	980; 0 (0–0)	760; 630 (310–980)	450; 110 (60–400)
Craniotomy	33	19 ± 21*	7 ± 9*	12 ± 16*	33,200; 21,410 (12,210-42,430)*	21,790; 11,900 (5690-26,650)*	7200; 4530 (4520-9060)*	1300; 970 (610–1750)*	1890; 1080 (500–2750)*
Decompressive craniectomy	24	28 ± 27*	11 ± 9*	17 ± 21*	49,750; 41,970 (26,400-68,830)*	34,370; 26,530 (14,120-50,400)*	8880; 8240 (4530-10,500)*	1840; 1880 (1110-2310)*	3230; 2850 (1290-4940)*
Extracranial surgery	65	12 ± 14*	2 ± 6	10 ± 12*	19,960; 13,900 (10,740-24,630)*	11,620; 6190 (3350-13,510)	5010; 3350 (3160-6490)*	1250; 1190 (750–1680)*	820; 310 (130–1070)
No extracranial surgery	421	7 ± 13	2 ± 5	6 ± 9	10,680; 4130 (2610-10,050)	7320; 2500 (1430-6400)	950; 0 (0–0)	770; 610 (310–970)	630; 110 (60–530)
In hospital mortality			*	*			*		*
Yes	60	7 ± 9	4 ± 6	3 ± 6	17,250; 9020 (6540-22,550)	10,790; 4330 (2670-14,540)	2320; 0 (0–4520)	980; 840 (640–1160)	1490; 910 (240–1940)
No		8 ± 13	2 ± 5	7 ± 10	11,170; 4530 (2640-11,890)	7490; 2500 (1430-6740)	1380; 0 (0–0)	820; 640 (310–1070)	530; 100 (60–420)
GOSE 6 months		*	*	*	*	*	*	*	*
1	73	9 ± 13	4 ± 7	4 ± 10	18,240; 8960 (5860-21,560)	11,890: 4520 (2670-13,520)	2370; 0 (0–4520)	980; 820 (570–1200)	1510; 970 (240–1960)
2/3	17	30 ± 29	7 ± 9	23 ± 21	36,190; 17,260 (12,290–48,500)	26,570; 13,010 (5420-34,890)	4710; 3720 (0–7070)	1850; 1750 (1320-2260)	2060; 1460 (220–4280)
4	23	8 ± 8	2 ± 6	6 ± 6	13,160; 7940 (2890-15,700)	8420; 2890 (1620-8270)	1760; 0 (0–3250)	1180; 1040 (270–1800)	670; 120 (60–460)
5	25	9 ± 8	2 ± 3	7 ± 6	13,080; 10,150 (3840-15,130)	8180; 5140 (2220-11,600)	1930: 0 (0–1820)	900; 830 (520–1140)	730; 180 (70–920)
6	38	7 ± 8	1 ± 2	7 ± 7	10,480; 5350 (3330-13,220)	6210; 2790 (1370-6430)	1810; 0 (0–3160)	1000; 880 (530–1190)	370; 80 (60–370)
7	110	7 ± 9	1 ± 5	5 ± 7	9100; 4010 (2780-9550)	6130; 2030 (1430-5840)	840; 0 (0–0)	770; 650 (370–980)	410; 80 (60–360)
8	131	4 ± 4	0 ± 1	4 ± 4	5780; 3210 (2310-7260)	3560; 1880 (1180-4570)	670; 0 (0–0)	560; 410 (270–780)	220; 70 (60–200)

Values are reported as: mean ± SD or mean; median (IQR 25–75)

Favourable and unfavourable were defined as GOSE 5–8 and GOSE 1–4 respectively. *AIS*, abbreviated injury scale; *CT scan*, computed tomography scan; *GCS*, Glasgow Coma Score; *GOSE*, Glasgow Outcome Score–Extended; *ICU*, intensive care unit; *SAH*, subarachnoid haemorrhage

^*^p value < 0.05: *p* values were derived from ANOVA for continuous characteristics. The *p* value assessed compatibility with the null hypothesis of no differences in mean values between row categories. Costs were rounded to the nearest ten euros

A total of 126 patients (27.2%) received a surgical intervention, of which 67 intracranial (13.8%) and 65 extracranial (13.4%). Intracranial surgery was significantly more common in more severely injured TBI subgroups (6.2% for mild, 34.9% for moderate and 35.9% for severe TBI) (Table 2).

### In-hospital costs

Mean in-hospital costs were €11,918. €7896 was related to admission (66%), €1493 to surgery (13%) and €1042 to other (9%) (Table 2). Costs related to radiology (7%) and laboratory (5%) were smaller contributors. Average in-hospital costs were €7795 for mild, €20,207 for moderate €26,595 for severe and €26,349 for very severe TBI patients (Fig. 2). Presence of intracranial abnormalities on the first CT scan nearly doubled total in-hospital costs (€15,783 vs. €8238). Intracranial surgery or ICP monitoring quadrupled the costs (respectively €36,866 vs. €7928 and €47,255 vs. €8748). Patients with a decompressive craniectomy (€49,754), ‘regular’ craniotomy (€33,195) or extracranial surgery (€19,957) were also more expensive compared with non-surgically treated patients. Patients with a 6-month GOSE score of 8 showed the lowest in-hospital costs of € 5774, while patients with a GOSE score of 2/3 showed costs of €36,190.

## Discussion

The current study found substantial in-hospital healthcare consumption and high in-hospital costs for hospitalised TBI patients, even after mild TBI. Both length of stay and in-hospital costs increased with TBI severity and presence of intracranial abnormalities and extracranial injuries. The most important cost drivers were admission and surgical intervention. Patients from all TBI severity categories were able to achieve full recovery, even after sustaining very severe TBI. Nonetheless, mortality and unfavourable outcome rates were high and the majority of patients reported remaining deficits or disabilities after 6 months.

### Study cohort

The predominance of male gender, injury mechanisms (road traffic accidents and falls) and distribution of TBI severity were in accordance with recent literature [7, 15, 29, 33]. The mean age of 56 years was rather high compared to earlier research [7], but matched changing epidemiological patterns [32]. The number of intracranial CT abnormalities in mild TBI patients was higher compared with that in literature (45.2% vs. 16.1%) [26]. This is likely caused by different inclusion criteria (hospital admission after TBI vs. ED presentation with head CT after suspected TBI) and differences in accuracy between central and local radiological reading [73]. The number of patients with major extracranial injury (AIS ≥ 3) and pupillary abnormalities was also higher compared with that in literature [72, 77] and the overall CENTER-TBI Core study cohort [59]. These factors, with other factors like comorbidities and use of anticoagulants, could have negatively influenced patient outcome and/or increased the reported in-hospital healthcare consumption and in-hospital costs in this study.

### Patient outcome

Mortality rates were generally high, but difficult to compare with other studies due to methodological differences [16, 32, 51]. One meta-analysis reported higher ‘all time point’ mortality rates for patients of all TBI severities [38], while other studies showed lower mortality rates for mild TBI [10], moderate TBI [16] and severe TBI [51, 58]. Favourable outcome (6-month GOSE) rates were generally higher in literature [39, 51, 16]. Differences in patient outcome can largely be explained by patient related factors that are known to be associated with worse outcome. Such factors include higher age, higher injury severity, poorer initial neurologic condition and higher TBI severity (defined by GCS) and are reported above average in our cohort [28, 38, 71]. For instance, the inclusion of patients with a GCS = 3 and/or bilateral pupillary abnormalities influences the comparison of patient outcome, as they are typically excluded in literature because of their often-perceived dismal prognosis [65]. Even the most severely injured patients that were able to achieve favourable outcome and even full recovery, although rarely, has been reported previously [71].

The increase in mortality rates (12.3 to 15%) and data on persisting deficits and disabilities after 6 months confirm the need for increased vigilance and attention for rehabilitation or long-term care opportunities. Sustained health problems after TBI have also been reported by long-term follow up studies [21, 42, 52, 74], some reporting deterioration between 5 and 10 years [17], others reporting remaining functional limitations up to 20 years after moderate and severe TBI [3]. Long-term impairments are not limited to severe TBI, but are also reported after mild TBI [14, 68]. Despite the short 6-month follow-up, our results support statements that consider TBI to be an acute injury resulting into a chronic health condition that requires continued care for most patients. TBI should therefore be addressed as such by healthcare providers, researchers and policymakers [60, 79].

### Length of stay

Healthcare consumption in terms of length of stay and surgical intervention was substantial. However, when comparing our overall results to numbers for patients (age < 65) from Canada, our mean LOS (days) was shorter for all patients (8 vs. 13), for patients with mild TBI (6 vs. 9) and severe TBI (15 vs. 22) but similar for moderate TBI (14 vs. 14) [62]. Median LOS was also shorter for mild TBI (3 vs. 9), moderate TBI (7 vs. 11) and severe TBI (7 vs. 12) compared with recent numbers from England and Wales [29]. In a review on hospital costs for severe TBI patients, total LOS ranged between 10 and 36.8 days and ICU LOS between 7.9 and 25.8 days [69]. The large ranges are exemplary for the existing variation, that is, primarily caused by patient case-mix and treatment-related factors [40]. Several factors that we found to be associated with an increased total LOS were also mentioned in literature: lower GCS, higher TBI severity and the presence of extracranial injury [13, 62], ICP monitoring [46, 61] and decompressive craniectomy [27, 53].

There were several exceptions. For instance, the most severely injured TBI patients were sometimes admitted to the ward because of treatment limiting decisions shortly after presentation [50]. This could explain the lower LOS and lower in-hospital costs for very severe TBI patients and patients with two non-reacting pupils. Similarly, some mild TBI patients could have been admitted to the ICU because of (suspected) deterioration or over-triage or non-TBI related issues such as age, comorbidities, and concomitant extracranial injuries [6, 36].

### In-hospital costs

The median costs and interquartile range indicate that costs were skewed by a small group of patients with very high costs. The reported costs were generally similar to available literature. One Dutch study reported that the direct and indirect costs for all TBI patients were €18,030 [56]. Costs were higher for Dutch patients with severe TBI (range €40,680–€44,952), but these costs included rehabilitation and nursing home costs [55]. A recent systematic review reported median in-hospital costs per patient with severe TBI of €55,267 (range €2130 to €401,808) [69]. Mean hospital and healthcare charges for TBI in the USA were $36.075 and $67.224 respectively [2, 35]. Differences between studies could be explained by variation, methodological heterogeneity, differences in case mix, but also by geographical location. For example, healthcare expenditures in the USA are generally double of other high-income countries due to prices of labour, goods, pharmaceuticals and administrative costs, while healthcare utilisation was similar [45]. These issues are also reported in non-TBI literature [12, 47].

As in other studies, the main cost drivers in this current study were LOS and/or admission (66%), surgery (12%), radiology (7%), labs (4%) and other costs (11%) [2, 41, 81]. In-hospital costs were generally higher for the more severely injured patients [35, 41], with a lower GCS [24, 41, 48, 63, 69] or pupillary abnormalities [70]. Higher costs were related to an increased healthcare consumption with longer LOS [2, 48], specialised intensive care unit (ICU) treatment [2] and a more frequent use of ICP monitoring [37, 61, 81] and surgical procedures [41, 70, 80]. The presence of TBI normally increases the LOS of general admissions [62], but extracranial injury and higher overall injury severity in addition to TBI also contributed to higher in-hospital healthcare consumption and in-hospital costs [13, 57, 80]. It is however impossible to distinguish costs related to extracranial injury from costs related to TBI because these costs are too intertwined.

Compared with the hospital costs for other diseases in the Netherlands, the in-hospital costs for TBI patients were high, especially when TBI severity increased. The hospital costs for patients with ischaemic stroke (€5.328) [8], transient ischaemic attack (€2.470) [8], appendicitis (€3700) and colorectal cancer (€9.777–€19.417) [20] were lower, while costs were higher for patients with non-small cell lung cancer (€33.143) [67] or patients receiving extracorporeal life support treatment (€106.263) [44].

### Strengths and limitations

The accurate calculation of in-hospital healthcare consumption and in-hospital costs of a large prospective multicentre cohort is a strength of the current study. There are also several limitations. The GCS is usually used to determine TBI severity [7], but its general applicability as a severity measure is also criticised [5]. The GCS could have been influenced by intoxication, pharmacological sedation, prehospital intubation, extracranial injury and could thereby have over- and underestimated injury severity [54]. This could have influenced study results. In a similar way, patient outcome was measured by using in-hospital mortality and GOSE. Critics state that the GOSE insufficiently accounts for the multidimensional nature of TBI outcome [32]. Unfortunately, earlier reported problems with acquiring the disease related health related quality of life outcome measure QOLIBRI resulted in too many missing data points to be useful for this manuscript [70]. Another limitation is the short-term follow-up because it is known that patient outcome and costs can change over time [17, 60, 79]. TBI patients that visited the ER but did not require hospitalisation were not included in this study. A precise calculation and comparison of costs was therefore not possible. Costs of these patients are expected to be substantially lower compared with those of admitted patients since important cost drivers (admission and surgery) are not applicable. Following the unit costs in Supplement 1 (ER, imaging, labs), the average costs are likely to be somewhere between €500 and €1.000. A reduction in number of admitted mild TBI patients, when safe and possible, might result in substantial cost savings, especially since its incidence is high.

The direct costs of TBI (all consumed resources within the health-care sector) are generally considered to be smaller than the indirect costs (loss of productivity and intangible costs) [22, 32, 43]. Because of the focus on in-hospital costs, our study results dramatically underestimate the exact total costs related to TBI [34, 56, 66]. The reported in-hospital costs are also likely to be an underestimation, despite our accurate calculations. More accurate numbers could be achieved by using hospitals’ actual cost prices, rather than approximations from guidelines or governmental organisations. These numbers were unfortunately unavailable. Including an accurate complete cost overview is however essential for future cost-effectiveness studies [11, 34, 48, 66].

Future TBI research initiatives should include the combination of long-term outcome and complete economic perspective, because this can improve the objectivity of future treatment decision-making. When striving for cost-effectiveness, people should however not forget the individual aspects of care and the social utility of providing care for severely injured patients [25].

## Conclusion

Hospitalised TBI patients show substantial in-hospital healthcare consumption and high in-hospital costs, even in patients with mild TBI. These costs are likely to be an underestimation of the actual total costs after TBI. Although patients from all TBI severity categories were able to achieve full recovery, mortality and unfavourable outcome rates were high and increased with TBI severity, intracranial abnormalities, extracranial injury and surgical intervention. Future studies should focus on the long-term effectiveness of treatments in relation to a complete economic perspective.

## Electronic supplementary material

ESM 1 (DOCX 20 kb)

## Abbreviations

CRF: Case report form
CT: Computed tomography
GCS: Glasgow Coma Score
GOSE: Glasgow Outcome Score–Extended
ICP: Intracranial pressure
ICU: Intensive care unit
LOS: Length of stay
TBI: Traumatic brain injury
