# Supplementary material for: Functional outcome, in-hospital healthcare consumption and in-hospital costs for hospitalised traumatic brain injury patients: a Dutch prospective multicentre study
Source: Acta Neurochir (Wien). 2020 May 14;162(7):1607–18. doi: 10.1007/s00701-020-04384-9 (PMC7295836; doi:10.1007/s00701-020-04384-9)
Supplement: Supplementary file 1 — (DOCX 20 kb) [file 701_2020_4384_MOESM1_ESM.docx]

**Supplement 1**

| **Health care unit** | **Cost category** | **Value (€)** | **Reference** | **Source** | **Value (2017)** | **Remark** |
| --- | --- | --- | --- | --- | --- | --- |
| **Transportation:** |  |  | | | | |
| Ambulance | Other | 613 | 2014 | Guideline^23^ | 627 | Costs for all immediate transportations |
| Mobile Medical Team (MMT) involvement | Other | 3,424 | 2011 | Multiple sources | 3,715 | Per unit. See * for calculation |
| **Admission:** |  |  |  |  |  |  |
| ER visit | Admission | 259 | 2014 | Guideline^23^ | 264 | Per visit |
| ICU admission | Admission | 2,148 | 2017 | NZA prices^83^ | 2,148 | Costs per day (category used: medium heavy) |
| High care admission | Admission | 561 | 2017 | NZA prices^83^ | 562 | Costs per day |
| Ward admission (general/academic hospital) | Admission | 443/642 | 2014 | Guideline^23^ | 453/657 | Costs per day |
| In-hospital consultations | Admission | 85-255 | 2017 | NZA prices (DIS)^82^ | 85-255 | Involvement of other specialists irrespective of reason/diagnosis. Counted as one visit. |
| Paramedical (such as physiotherapy, vocational therapy) | Admission | 30-33 | 2014 | Guideline^23^ | 31-34 | Price per visit. Counted as 1 if involved. |
| Outpatient clinic visit (general/academic hospital) | Other | 80/163 | 2014 | Guideline^23^ | 82/166 | Per visit, related to injury |
| **Surgical intervention**** |  |  | | | | |
| - Craniotomy | Surgery | 4,515 | 2017 | NZA prices (DIS)^82^ | 4,515 | Brain surgery, without admission costs |
| - Decompressive craniectomy | Surgery | 4,515 | 2017 | NZA prices (DIS)^82^ | 4,515 | Brain surgery, without admission costs |
| - Replacement of bone flap | Surgery | 3,720 | 2017 | NZA prices (DIS)^82^ | 3,720 | Brain surgery, without admission costs |
| - External ventricular drainage | Surgery | 3,720 | 2017 | NZA prices (DIS)^82^ | 3,720 | Brain surgery, without admission costs |
| - Placement of ICP monitor | Surgery | 3,720 | 2017 | NZA prices (DIS)^82^ | 3,755 | Brain surgery, without admission costs |
| - Burr hole drainage | Surgery | 3,720 | 2017 | NZA prices (DIS)^82^ | 3,720 | Brain surgery, without admission costs |
| - Extracranial surgeries | Surgery |  | 2017 | NZA prices (DIS)^82^ |  | Extracranial surgeries, without admission costs |
| **Imaging studies** |  |  | | | | |
| - CT brain | Radiology | 131,90 | 2017 | NZA prices^83^ | 132 | Per unit |
| - CT spine | Radiology | 136.12 | 2017 | NZA prices^83^ | 136 | Per unit |
| - MRI brain | Radiology | 272,32 | 2017 | NZA prices^83^ | 272 | Per unit |
| - Other imaging studies | Radiology | 37.23-253.01 | 2017 | NZA prices^83^ | 37-253 | Different studies, price per unit. |
| **Laboratory studies***** |  |  | | | | |
| -Arterial blood gas | Laboratory | 10 | 2017 | NZA prices^83^ | 10 | Per unit |
| -Hematology | Laboratory | 8 | 2017 | NZA prices^83^ | 8 | Per unit |
| -Chemistry | Laboratory | 51 | 2017 | NZA prices^83^ | 51 | Per unit |
| -Coagulation | Laboratory | 15 | 2017 | NZA prices^83^ | 15 | Per unit |
| -Blood culture | Laboratory | 28.93 | 2017 | NZA prices^83^ | 29 | Per unit |
| -Other cultures (urine, CSF, sputum etc) | Laboratory | 13.20 | 2017 | NZA prices^83^ | 13 | Per unit |
| **Blood products** |  |  |  |  |  |  |
| -Erythrocytes/thrombocyten/plasma | Laboratory | 216/522/186 | 2014 | Guideline^23^ | 221/534/190 | Per unit |
| -Prothrombincomplex | Laboratory | 210.94 | 2017 | Medicijnkosten.nl | 211 | Per 10ml/250IE. If amount unknown: 20ml |

**Caption:**

Supplement 1 shows a detailed overview of all used unit costs and corresponding sources.

**Legend:**

*Own calculation: National total costs for 2011: 21.3 million EUR (source: <https://www.recht.nl/rechtspraak/uitspraak/?ecli=ECLI:NL:CBB:2015:428>; accessed 06-09-2019. National total use: 6220 units (https://www.ambulancezorg.nl/static/upload/raw/1aa59bb7-a8e0-48e5-897e-06f9703a7e3d/ambulances-in-zicht-2015.pdf; accessed 06-09-2019). Unit price =€3,424 (21,300,000/6.223).
**Surgical interventions are classified according to the most frequently recorded operation in the database (www.opendisdata).
***Due to the major variation in laboratory studies only the most commonly performed studies were counted. Prices are calculated based on the NZA prices of individual studies.

**References**:

23. Hakkaart-van Roijen L vdLN, Bouwmans CAM, Kanters TA, Tan SS. Kostenhandleiding: Methodologie van kostenonderzoek en referentieprijzen voor economische evaluaties in de gezondheidszorg. Zorginstituut Nederland. Geactualiseerde versie 2015. https://www.zorginstituutnederland.nl/binaries/zinl/documenten/publicatie/2016/02/29/richtlijn-voor-het-uitvoeren-van-economische-evaluaties-in-de-gezondheidszorg/Richtlijn+voor+het+uitvoeren+van+economische+evaluaties+in+de+gezondheidszorg+%28verdiepingsmodules%29.pdf. Accessed 30 Sep 2019.

83. Zorgautoriteit Nederland. Tarieventabel DBC-zorgproducten en overige producten - per 1 januari 2012 (PUC_12710_22). https://puc.overheid.nl/nza/doc/PUC_12710_22/1/. Accessed Accessed 29 Sep 2019

82. Zorgautoriteit Nederland. Open data van de Nederlandse Zorgautoriteit http://www.opendisdata.nl. Accessed 29 March 2019
